# Measurement of macular structure-function relationships using spectral domain-optical coherence tomography (SD-OCT) and pattern electroretinograms (PERG)

**DOI:** 10.1371/journal.pone.0178004

**Published:** 2017-05-17

**Authors:** Keunheung Park, Jinmi Kim, Jiwoong Lee

**Affiliations:** 1 Department of Ophthalmology, Pusan National University College of Medicine, Busan, Korea; 2 Department of Biostatistics, Clinical Trial Center, Biomedical Research Institute, Pusan National University Hospital, Busan, Korea; 3 Biomedical Research Institute, Pusan National University Hospital, Busan, Korea; Bascom Palmer Eye Institute, UNITED STATES

## Abstract

**Background:**

Retinal ganglion cell (RGC) death is a common cause of loss of vision during glaucoma. Pattern electroretinogram (PERG) is an objective measure of the central retinal function that correlates with macular GCL thickness. The aim of this study is to determine possible relationships between the N95 amplitude of pattern electroretinogram (PERG_amp_) and macular ganglion cell/inner plexiform layer thickness (GCIPLT).

**Methods and findings:**

This was a retrospective and comparative study including 74 glaucoma patients (44 early stage and 30 advanced stage cases) and 66 normal control subjects. Macular GCIPLT was measured using Cirrus spectral domain-optical coherence tomography. Standard automated perimetry and pattern ERGs were used in all patient examinations. Three types of regression analysis (broken stick, linear regression, and quadratic regression) were used to evaluate possible relationships between PERG_amp_ and GCIPLT. Correlations between visual field parameters and GCIPLT were evaluated according to glaucoma severity. The best fit model for the relationship between PERG_amp_ and GCIPLT was the linear regression model (r^2^ = 0.22; *P* < 0.001). The best-fit model for the relationship between visual field parameters and GCIPLT was the broken stick model. During early glaucoma, macular GCIPLT was positively correlated with PERG_amp_, but not with visual field loss. In advanced glaucoma, macular GCIPLT was positively correlated with both PERG_amp_ and visual field loss.

**Conclusions:**

PERG_amp_ was significantly correlated with macular GCIPT in early glaucoma patients, while visual field performance showed no correlation with GCIPLT. PERG_amp_ can therefore assist clinicians in making an early decision regarding the most suitable treatment plan, especially when GCIPLT is thinning with no change in visual field performance.

## Introduction

The main characteristic of glaucoma is loss of retinal ganglion cell axons, which typically leads to optic neuropathy. Retinal ganglion cell (RGC) death is a common cause of loss of vision during glaucoma and most optic neuropathies. Stress in the cellular and molecular environment that exceeds the survival capacity of RGCs can lead to progressive damage to ganglion cells and their fibers. This damage can induce retinal dysfunction, as revealed by flash electroretinogram (FERG) or pattern electroretinogram (PERG) recordings [1–4].

Macular ganglion cell loss has been detected during early experimental glaucoma [5]. Nagaraju et al. [6] reported that RGC dysfunction can occur with an increase in intraocular pressure (IOP); changes in PERG could be an effective tool to noninvasively assess the susceptibility of RGCs to increases in IOP. Previous studies also reported that as many as half of all RGCs, and their axons, may be lost before loss of visual function is detected [7–9]. Thus, early diagnosis is especially important in the treatment of glaucoma. Before ganglion cells are severely damaged or destroyed, glaucomatous damage may be reversible. Ventura et al. [10] reported that in early glaucoma, a reduction of IOP restored both early visual field loss and PERG amplitudes.

Loss of RGCs leads to atrophy of the ganglion cell layer (GCL). In eyes with pre-perimetric glaucoma, the macular GCL shows severe thinning [11]. The macular GCL is thick and multilayered, with approximately 50% of RGCs concentrated in this region. The axons of macular RGCs, and of some peripheral RGCs outside of the macula, also pass through the macular area to reach the optic nerve head. Study of the macular GCL and retinal nerve fiber layer (RNFL) may therefore be appropriate to evaluate RGC loss. Recent studies using spectral domain-optical coherence tomography (SD-OCT) reported that loss of thickness of the RNFL, GCL, and inner plexiform layer (IPL) combined, termed the ganglion cell complex, occurs during early glaucomatous damage [12,13].

PERG signals are comprised of positive P50 and negative N95 components. The positive P50 component is always affected by retinal/macular dysfunction, while the late negative N95 component is mainly affected by optic nerve diseases [14]. A previous study reported that PERG was generated more from the inner layers of the retina than was the FERG [15]. Thus, PERG is an objective measure of the central retinal function that correlates with macular GCL thickness.

A previous study reported that PERG loss was greater than circumpapillary RNFL thinning [16]. However, there has been no report of a correlation between electroretinogram (ERG) findings and macular GCL. Therefore, the purpose of this study was to determine if there is a correlation between PERG amplitude macular GCIPL thickness.

## Materials and methods

This was a retrospective and comparative study, performed at Pusan National University Hospital (Busan, Korea) from July 1, 2015 to August 31, 2016. The study was performed in accordance with the tenets of the Declaration of Helsinki and was approved by the Institutional Review Board (IRB) of Pusan National University Hospital. The institutional review board waived consent from patients because this is a retrospective, anonymous study.

Participants were ≥ 18 years of age, and were either healthy or had been previously diagnosed with open-angle glaucoma. Glaucoma patients were further classified as having early [mean deviation (MD) ≥ -6 dB] or moderate/advanced (MD < -6 dB) glaucoma. Patients were excluded if they met any one of the following exclusion criteria: corneal scarring, media opacities, anterior segment dysgenesis, past chronic steroid use, history of diabetic retinopathy, previous intraocular surgery (except uncomplicated cataract surgery), a refractive error outside the -6.00 to +6.00 diopters range, and any abnormality other than glaucoma. When both eyes were involved, one eye was chosen randomly.

A complete medical history was taken from the participants, all of whom underwent an ophthalmic examination by a glaucoma specialist. Clinical data included best-corrected visual acuity, and results of Goldmann applanation tonometry, slit-lamp examination, gonioscopy, pachymetry (Pachmate; DGH Technology, Exton, PA, USA), and fundus examination with a dilated pupil. Age, sex, and spherical equivalent refractive error (ARK-510A; NIDEK, Hiroishi, Japan) were also recorded for all participants.

### PERG measurements

The PERG measurements were recorded using a commercial ERG system (RETIport 32; Roland Instruments, Brandenburg, Germany) that conformed to the International Society of Clinical Electrophysiology and Vision (ISCEV) standards of 2012 [17]. All patients were prepared by appropriate optical correction without dilation of the pupils. Background illumination remained constant during the examination, at approximately 50 lux. Ground electrodes were placed on the forehead, and reference electrodes were placed on the skin near the ipsilateral outer canthus. An active electrode (H-K loop; Avanta, Ljubljana, Slovenia) was placed in the lower conjunctival sac of each eye. Loops were folded so that the contact windows on the insulated wire were positioned on the bulbar conjunctiva. The mean width and height of the stimulus field were both 15 ± 3°, with a check size of 0.8 ± 0.16°. The contrast between the black and white squares was 98%. The mean luminance was 85 candela/m^2^. The pattern ERGs were obtained as binocular recordings at a reversal rate of 2 ± 0.4 Hz, and at least 100 artifact-free sweeps were collected and averaged.

The P50 amplitude was measured from the trough of N35 to the peak of P50. The N95 amplitude (PERG_amp_) was measured from the peak of P50 to the trough of N95. The implicit times of the P50 and N95 were also measured.

### Visual field test

Perimetry involved Swedish interactive threshold algorithm 24–2 of the Humphrey Visual Field Analyzer 750i instrument (Carl Zeiss Meditec, Dublin, CA, USA). Reliable visual field tests were defined as a false positive rate < 15%, false negative rate < 33%, and fixation loss < 20%. Normal control subjects had a glaucoma hemifield test (GHT) within normal limits and their MD and pattern standard deviation (PSD) were within 95% of the normal population. Glaucomatous visual fields were those that met at least one of the following criteria: a GHT outside normal limits and a PSD probability outside of 95% of the normal population.

**Fig 1:**
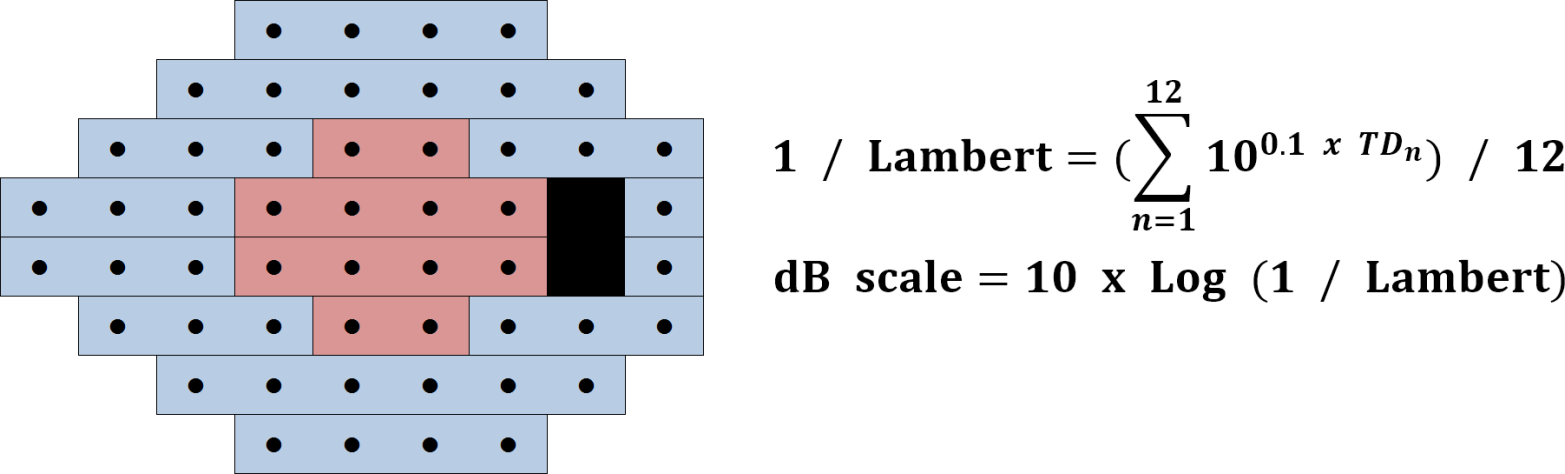
Calculation of central visual field sensitivity. Total deviation values (TD_n_) of central 12 points (out of 54 points) are unlogged, averaged, and finally logged again to transform back to the decibel scale.

Visual field total deviation values were recorded at all 52 testing points, among which the 12 most central points, i.e. those corresponding to the macular area, were selected. The selected values were unlogged, averaged, and finally logged again to transform them back to a decibel scale, termed the central visual field total deviation (VF_center_; Fig 1).

### SD-OCT

The Cirrus SD-OCT instrument (Carl Zeiss Meditec) (version 6.0 software) was used to measure ganglion cell/inner plexiform layer thickness (GCIPLT). After pupil dilation using 0.5% tropicamide and 0.5% phenylephrine, the Cirrus SD-OCT instrument was used to acquire a single macular scan (200 × 200 macular cube scan protocol) of each eye studied. The GCA algorithm automatically segmented the GCIPL and RNFL and calculated the thickness of the macular GCIPL and RNFL within a 14.13 mm^2^ elliptical annulus area centered on the fovea. Average, minimum, and six sectoral (superotemporal, superior, superonasal, inferonasal, inferior, and inferotemporal) GCIPLT and retinal nerve fiber layer thickness (RNFLT) values were obtained, and the average values were used in the statistical analyses. All of the included SD-OCT scans had a signal strength of at least six.

### Statistical analyses

Three statistical regression models, namely broken stick, linear regression, and quadratic regression models, were used to evaluate macular structure-function relationships. The broken stick model is a nonlinear statistical model consisting of two linear regression lines with tipping point. An initial estimate of the tipping point was determined using the Davies’ test [18]; then, segmental regression analyses were performed starting with this initial tipping point. The final tipping point, and the two slopes of the broken stick model, were determined using segmental regression analyses to reduce error. The R language (http://www.R-project.org) and segmented R library were used for the Davies’ test and segmented regression analyses [19]. Linear and quadratic regression models were determined using the following equations: linear, y = a + bx and quadratic, y = a + bx + cx^2^. To compare the fitness of the three regression models, we used the Akaike information criterion (AIC). AIC estimated the quality of each model relative to the other models. Among a given set of candidate models, the best model will have the lowest AIC value. Pearson’s or Spearman’s correlation analyses were also used to compare the macular GCIPLT, RNFLT, PERG amplitudes and visual field parameters [visual field MD (VF_MD_) and VF_center_] according to the severity of glaucoma.

The normality of the data distribution was checked using the Kolmogorov-Smirnov test. To compare parameters between normal subjects and glaucoma patients, one-way analysis of variance, or the Kruskal-Wallis test, was used according to the normality of the data. The chi-squared test was used for the categorical variables. A *P* -value < 0.05 was considered statistically significant.

## Results and discussion

In total, data from 66 normal control subjects and 74 glaucoma patients (44 early and 30 early advanced stage) were included (63 males and 77 females). The patient demographics are summarized in Table 1. No patient had diabetic or hypertensive retinopathy, although some had a past history of diabetes or hypertension. There was no significant difference in sex, axial length, or spherical equivalent between the groups, but there were differences in age and central corneal thickness (*P* = 0.004 and *P* = 0.029, respectively).

**Table 1:** Demographic characteristics of the healthy and glaucomatous subjects.

	Healthy (n = 66)	Glaucoma (n = 74)		*P* value
		Early (n = 44)	Advanced (n = 30)	
Age (year)	48.3 ± 14.2	49.5 ± 14.6	58.4 ± 12.9	0.004^a^
Female/Male (number of patient)	41 / 25	23 / 21	13 / 17	0.209^b^
Diabetes (number of patient)	4	5	5	0.258^b^
Hypertension (number of patient)	9	10	11	0.038^b^
Axial length (mm)	24.26 ± 1.45	24.42 ± 1.77	24.31 ± 1.53	0.897^c^
Spherical equivalent (diopters)	–1.76 ± 2.80	–1.74 ± 3.20	–1.31 ± 3.25	0.473^c^
Central corneal thickness (μm)	558.8 ± 32.7	544.6 ± 32.0	541.8 ± 35.5	0.029^a^
VF MD (dB)	–0.97 ± 1.49	–3.23 ± 1.64	–12.2 ± 5.87	<0.001^a^
VF PSD (dB)	1.83 ± 0.85	3.59 ± 2.13	9.76 ± 2.89	<0.001^c^
VF VFI (%)	98.56 ± 1.52	92.86 ± 12.14	67.87 ± 20.07	<0.001^c^
Mean PERG_amp_ (μV)	7.11 ± 2.20	5.30 ± 1.72	4.87 ± 1.78	<0.001^a^
Mean GCIPL thickenss (μm)	81.91 ± 6.12	74.59 ± 7.43	66.33 ± 8.56	<0.001^a^
Mean RNFL thickness (μm)	33.42 ± 3.62	29.52 ± 4.90	25.23 ± 5.30	<0.001^a^

VF MD visual field mean deviation, VF PSD visual field pattern standard deviation, VF VFI visual field visual field index, PERGamp pattern electroretinogram N95 amplitude (peak of P50 to the trough of N95), GC/IPL ganglion cell/inner plexiform layer, RNFL retinal nerve fiber layer

^a^ one-way ANOVA test

^b^ χ2 test

^c^ Kruskal-Wallis test

The mean PERGs_amp_ values were 7.11 ± 2.20 μV, 5.30 ± 1.72 μV, and 4.87 ± 1.78 μV (normal control subjects, early glaucoma patients, and advanced glaucoma patients, respectively) and were significantly different (*P* < 0.001). The mean GCIPLT values were 81.91 ± 6.12 μM, 74.59 ± 7.43 μM, and 66.33 ± 8.56 μM, and the mean RNFLTs were 33.42 ± 3.62 μM, 29.52 ± 4.90 μM, and 24.34 ± 1.62 μM (normal control subjects, early glaucoma patients, and advanced glaucoma patients, respectively). These two SD OCT parameters were significantly different between normal control subjects, early glaucoma patients, and advanced glaucoma patients (*P* < 0.001 for GCIPLT and *P* <0.001 for RNFLT, respectively).

**Fig 2:**
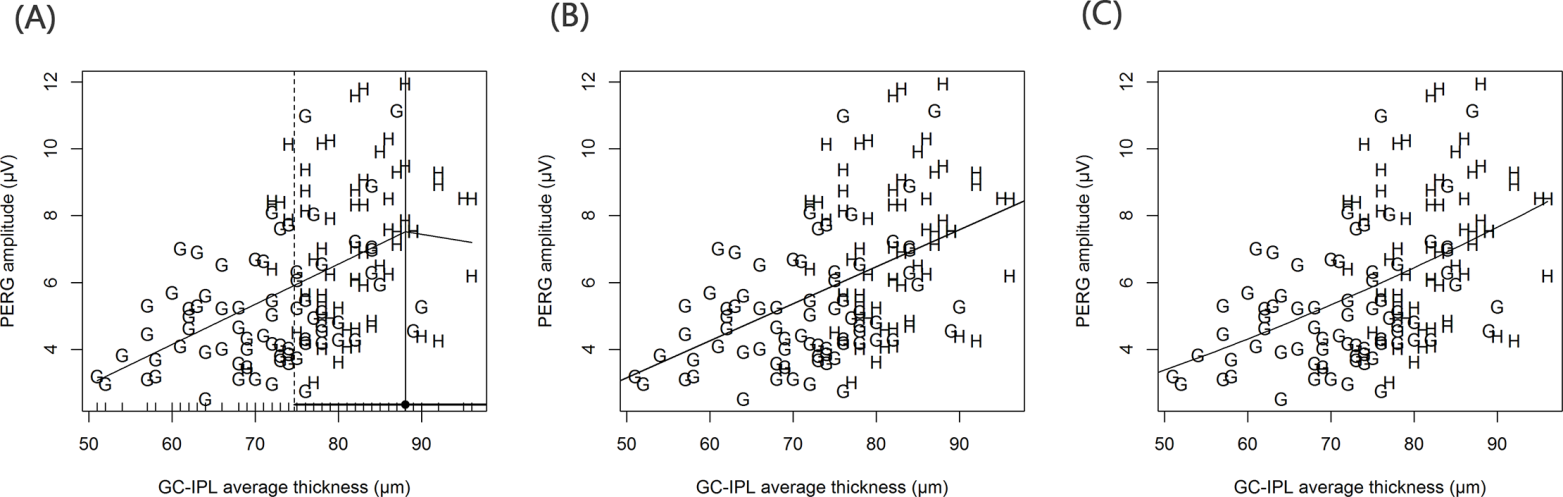
Scatter plots showing relationship between ganglion cell/inner plexiform layer (GCIPL) thickness and pattern electroretinogram N95 amplitude (PERG_amp_) in entire study sample. Three different regression models applied; (A) broken stick model (B) linear regression model (C) quadratic regression model. The best fit model for GCIPL thickness and PERG_amp_ was linear regression model (r^2^ = 0.220, *P* < <0.001, AIC = 588.7).

**Fig 3:**
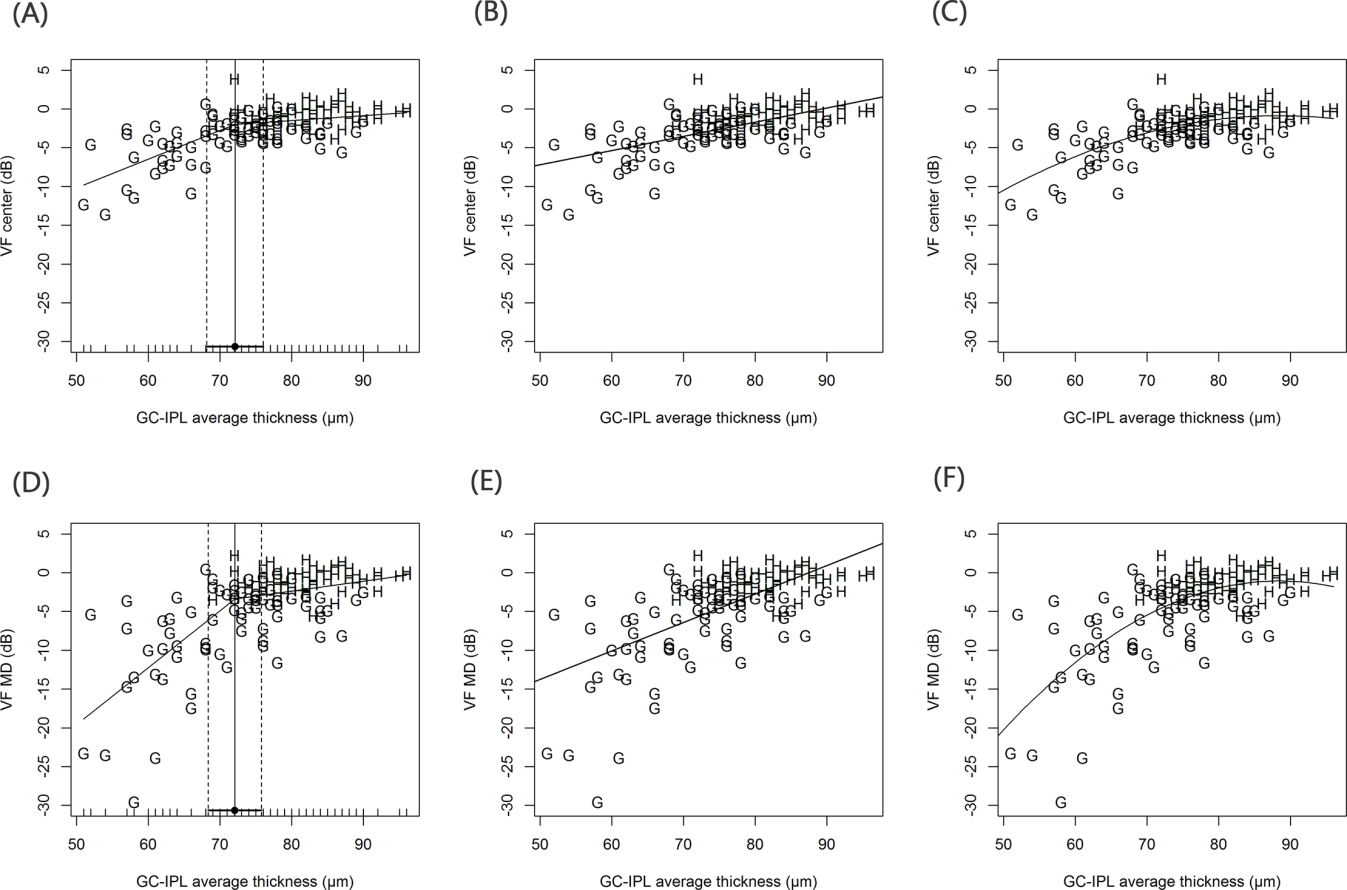
Scatter plots showing relationship of central visual field sensitivities (VF_center_) (A, B, C) or visual field mean deviation (VF_MD_) (D, E, F) with ganglion cell/inner plexiform layer (GCIPL) thickness in entire study sample. Three regression models were applied (broken stick model / linear regression mode / quadratic regression model respectively from left to right). For the GCIPL thickness and VF_center_, broken stick model was best fitted model with significant tipping point (Davies’ test *P* < 0.001) where the location was 71.9 μm. The broken stick model was best fitting model for the GCIPL thickness and VF_MD_ among the three different regression models. The tipping point was significant (Davies’ test *P* < 0.001) where the location was 72.1 μm. *VF*_*center*_ visual field centeral sensitivity, *VF*_*MD*_ visual field mean deviation, *GCIPL* ganglion cell/ inner plexiform layer.

The results of the regression analyses are summarized in Table 2. The best-fit model for the relationship between GCIPLT and PERG_amp_ was the linear regression model (r^2^ = 0.220, *P* < <0.001; AIC = 588.7). A scatterplot of the three regression models is shown in Fig 2. The broken stick model was the best-fit model (Table 2; Fig 3A, 3B and 3C) for the relationship between GCIPLT and VF_center_, with a significant tipping point (Davies’ test, *P* < 0.001) at 71.9 μM. When the GCIPLT was greater than the tipping point, the VF_center_ was unrelated to the GCIPLT (*P* = 0.050) and the slope was nearly zero (slope = 0.06). However, as the GCIPLT became thinner than the tipping point, the VF_center_ and GCIPLT became significantly correlated (*P* < 0.001) and the slope below the tipping point was steeper than the slope above the tipping point (slope = 0.37 vs. 0.06, respectively; *P* < 0.001). The relationship between GCIPLT and VF_MD_ was similar to that between GCIPLT and VF_center_ (Table 2; Fig 3D, 3E and 3F). Among the three different regression models, the broken stick model had the best-fit. The tipping point was statistically significant at 72.1 μM (Davies’ test, *P* < 0.001). The slopes below and above the tipping point were 0.74 and 0.13, respectively, and were significantly different from each other (*P* < 0.001).

**Table 2:** The relationship between ganglion cell/inner plexiform layer thickness and pattern electroretinogram amplitude or central visual field central sensitivities or mean deviation by regression models.

	Broken stick						Linear regression				Quadratic regression			
	Tipping point	Davies’ Test *P* value^a^	Slope 1^b^	*P* values	R^2^	AIC^d^	Slope	*P* value	R^2^	AIC^d^	Beta1	*P* value	R^2^	AIC^d^
			Slope 2^c^								Beta2			
			△Slope											
GCIPLT	88.0	0.934	0.12	<0.001	0.227	591.4	0.111	<0.001	0.220	588.7	0.043	0.841	0.221	590.6
vs.			–0.04	0.831							0.001	0.750		
PERG_amp_			–0.16	0.395										
GCIPLT	71.9	<0.001	0.37	<0.001	0.483	597.1	0.184	<0.001	0.389	616.3	1.157	<0.001	0.466	599.6
vs.			0.06	0.050							–0.007	<0.001		
VF_center_			–0.31	<0.001										
GCIPLT	72.1	<0.001	0.74	<0.001	0.512	773.8	0.366	<0.001	0.414	795.4	2.303	<0.001	0.495	776.6
vs.			0.13	0.058							–0.013	<0.001		
VF_MD_			–0.61	<0.001										

*VF*_*MD*_ visual field mean deviation; *VF*_*center*_ central visual field sensitivity; *PERG*_*amp*_ pattern electroretinogram N95 amplitude (peak of P50 to the trough of N95), *GCIPLT* ganglion cell/inner plexiform layer thickness

^a^ Davies’ test *P* value: the probability of that the tipping point is not statistically significant.

^b^ slope below the tipping point

^c^ slope above the tipping point

^d^ Akaike information criterion. The lower value means the better fitted model.

The correlations among macular GCIPLT, RNFLT, PERG_amp_, and visual field parameters (VF_center_ and VF_MD_) are summarized in Tables 3 to 5. In normal control subjects, there was no significant correlation between the structural and functional parameters (Table 3). However, in early glaucoma patients, macular GCIPLT was positively correlated with PERG_amp_, but not with VF_center_ or VF_MD_ (Table 4). In advanced glaucoma patients, PERG_amp_ was significantly correlated with macular GCIPLT. VF_MD_ showed a significant correlation with both macular GCIPLT and RNFLT, and VF_center_ showed a significant correlation with GCIPLT, but not with RNFLT (Table 5).

**Table 3:** Pearson’s correlation coefficient for structural and functional measures in normal subjects (n = 66 eyes).

	PERG_amp_	VF_MD_	VF_Center_	GCIPL thickness
VF_MD_	0.020			
VF_Center_	0.053	0.910^a^		
GCIPL thickness	0.195	0.204	0.165	
RNFL thickness	0.197	0.094	0.074	0.405^a^

*VF*_*MD*_ visual field mean deviation, *VF*_*center*_ central visual field sensitivity, *PERG*_*amp*_ pattern electroretinogram N95 amplitude (peak of P50 to the trough of N95), *GC/IPL* ganglion cell/inner plexiform layer, *RNFL* retinal nerve fiber layer

^a^*P* < 0.01

**Table 4:** Pearson’s or Spearman’s correlation coefficient for structural and functional measures in patients with early glaucoma (n = 44 eyes).

	PERG_amp_	VF_MD_	VF_Center_	GCIPL thickness
VF_MD_	–0.018^c^			
VF_Center_	–0.061^c^	0.853^b^		
GCIPL thickness	0.331^b^	–0.070^c^	0.052^c^	
RNFL thickness	–0.048^c^	0.198^c^	0.291^c^	0.413^a^^,^^c^

*VF*_*MD*_ visual field mean deviation, *VF*_*center*_ central visual field sensitivity, *PERG*_*amp*_ pattern electroretinogram N95 amplitude (peak of P50 to the trough of N95), *GC/IPL* ganglion cell/inner plexiform layer, *RNFL* retinal nerve fiber layer

^a^*P* < 0.01

^b^*P* < 0.05

^c^Spearman’s rho

**Table 5:** Pearson’s or Spearman’s correlation coefficient for structural and functional measures in patients with advanced glaucoma (n = 30 eyes).

	PERG_amp_	VF_MD_	VF_Center_	GCIPL thickness
VF_MD_	0.047^c^			
VF_Center_	0.079^c^	0.547^a^^,^^c^		
GCIPL thickness	0.505^a^	0.503^a^^,^^c^	0.431^b^^,^^c^	
RNFL thickness	0.459^b^	0.453^b^^,^^c^	0.313^c^	0.778^a^

*VF*_*MD*_ visual field mean deviation, *VF*_*center*_ central visual field sensitivity, *PERG*_*amp*_ pattern electroretinogram N95 amplitude (peak of P50 to the trough of N95), *GC/IPL* ganglion cell/inner plexiform layer, *RNFL* retinal nerve fiber layer

^a^*P* < 0.01

^b^*P* < 0.05

^c^Spearman’s rho

The main objective of this study was to determine if there were correlations between structural changes, represented by macular GCIPLT, and functional changes in RGCs, represented by PERG_amp_. Using regression analyses, the best-fit model for the relationship between GCIPLT and PERG_amp_ was the linear regression model. The best fit statistical model for the relationship between GCIPLT and VF_center_ was the broken stick tipping point model. In early glaucoma patients, macular GCIPLT showed a significant correlation with PERG_amp_, but not with visual field loss. In advanced glaucoma patients, macular GCIPLT was significantly correlated with both PERG_amp_ and visual field loss. Taken together, the results of the best fit regression models and correlation analyses according to glaucoma severity showed that substantial GCIPLT loss was necessary before visual field loss became detectable, while GCIPLT was positively correlated with functional loss, as measured by early PERG.

The pathophysiology of glaucoma primarily involves RGCs and their axons [20]. Approximately 50% of RGCs are concentrated within a 4.5 mm area at the center of the fovea [21]. PERG can index macular RGC function [22]. It has been suggested that GCIPLT thickness is the most important parameter when measuring glaucoma severity. Nakano et al. [11] reported that in pre-perimetric glaucoma patients, macular GCIPLT decreased more than macular RNFLT, and macular GCL thinning was more useful for detection of pre-perimetric glaucoma. Banitt et al. [23] reported that progressive RGC functional loss preceded structural loss by several years in suspected glaucoma patients. In a similar manner, the present study showed that PERG_amp_ was significantly correlated with macular GCIPLT, but not with visual field loss or macular RNFLT in early glaucoma patients. Furthermore, PERG_amp_ loss only correlated with macular RNFLT in advanced glaucoma patients.

It is possible that substantial GCL thinning is necessary before visual field loss is detectable. In the present study, the relationship between macular GCIPLT and visual field loss was best-fitted with the broken stick model, and the tipping point for VF_MD_ versus GCIPLT was 72.1 μM. Rapid visual field loss did not start until thinning of the GCL reached this tipping point. Consistent with this finding, Kim et al. [24] reported that the association of average GCIPLT with visual field macular sensitivity was not significant in pre-perimetric and early glaucoma patients. Harwerth et al. [9] reported that current perimetry regimens, with either white or monochromatic stimuli, were not useful for estimating ganglion cell loss until a substantial proportion of these cells had died. In their report, visual sensitivity losses were not detected until ganglion cell losses < 30–50%.

PERG has not always been found to be correlated with the results of visual field tests. Marx et al. [2] reported that glaucoma starts with subclinical panretinal damage of the ganglion cells, reflected in PERG but not necessarily in conventional visual field tests. Bach et al. [25] also reported that despite severe loss of PERG amplitude, the visual field remained normal because PERG preceded impairment of the ganglion cells. Quigley et al. [26] reported that it was necessary for half of the ganglion cells to be missing before visual field defects became detectable. In the present study, PERG_amp_ was significantly correlated with macular GCIPLT, but not with visual field parameters, in both early and advanced glaucoma patients. We hypothesize that this was because PERG measures the function of ganglion cells mainly concentrated at the foveal center, whereas measurable scotoma appears preferentially in the Bjerrum area during progression of glaucoma. PERG_amp_ may not correlate with visual field test results until substantial ganglion cell loss has occurred, especially in the central area.

Glaucoma is the leading cause of irreversible blindness worldwide [27,28] and early detection is key to preserving vision. In the present study, attenuation of PERG_amp_ started before thinning of the macular RNFL and loss of the visual field. Preservation of ganglion cells, and recovery of function, are possible at this early stage [29]. PERG_amp_ can therefore assist in the early diagnosis of glaucoma. We suggest that measuring PERG_amp_ is a valuable clinical option, especially in patients with early glaucoma and macular GCL thinning but with no detectable visual field changes. Furthermore, the reproducibility of PERG is sufficient for it to be a useful complementary clinical tool [30].

A limitation of this study was its retrospective nature. Clinicians should be careful when longitudinally applying their findings to individual subjects, because intrapatient variation in PERG_amp_ has not been established, and changes in PERG_amp_ can result from variations in measurement methods. Another limitation was that there is no standard international reference range for PERG measurements. Clinicians need to be aware that normal range of PERG_amp_ can be different from the data obtained in large population-based studies. The ISCEV has recommended that laboratories should establish normal values according to their own equipment and patient populations [17].

## Conclusions

In conclusion, PERG_amp_ was significantly correlated with macular GCIPLT in early glaucoma patients, but visual field test results showed no correlation with macular GCIPLT and PERG_amp_. Measurement of PERG_amp_ can therefore assist clinicians in making early decisions regarding effective and reliable treatment options, especially when the macular GCL is thinning but there is no change in the visual field test.

## Supporting information

S1 File The spreadsheet data file used in this study. (XLSX)

## Abbreviations

AIC: Akaike information criterion
BCVA: best corrected visual acuit
FERG: flash electroretinogram
GAT: Goldmann applanation tonometry
GCC: ganglion cell complex
GCIPLT: ganglion cell/inner plexiform layer thickness
GCL: ganglion cell layer
GHT: glaucoma hemifield test
IOP: intraocular pressure
IPL: inner plexiform layer
ISCEV: International Society of Clinical Electrophysiology and Vision
MD: mean deviation
ERG: pattern eletroretinograms
PERGamp: N95 amplitude of pattern electroretinogram
PSD: pattern standard deviation
RGC: retinal ganglion cell
RNFL: retinal nerve fiber layer
SD-OCT: spectral domain–optical coherence tomography
