# Correction: Measurement of macular structure-function relationships using spectral domain-optical coherence tomography (SD-OCT) and pattern electroretinograms (PERG)

**DOI:** 10.1371/journal.pone.0181390

**Published:** 2017-07-10

**Authors:** Keunheung Park, Jinmi Kim, Jiwoong Lee

## Notice of Republication

This article was republished on May 26, 2017, as the original S1 Dataset was published in error. Please download this article again to view the correct version.
